# Supplementary material for: Development and Validation of a Prognostic Model for Esophageal Adenocarcinoma Based on Necroptosis-Related Genes
Source: Genes (Basel). 2022 Nov 29;13(12):2243. doi: 10.3390/genes13122243 (PMC9778007; doi:10.3390/genes13122243)
Supplement: Supplementary file 1 [file genes-13-02243-s001.zip › Supplemental Table S1.pdf]

Supplemental Table S1. Clinical and pathologic information of EAC patients.

Variables	Number	Constituent Ratio（%）
Age（years）		
≤ 65	22	40.0
> 65	33	60.0
Gender		
Male	47	85.5
Female	8	14.5
T		
T1 / T2	26	47.3
T3 / T4	29	52.7
N		
N_0	17	30.9
N_+	38	69.1
M		
M_0	50	90.9
M_1	5	9.1
TNM staging		
I / II	28	50.9
III / IV	27	49.1
