# Supplementary material for: Development and Validation of a Prognostic Model for Esophageal Adenocarcinoma Based on Necroptosis-Related Genes
Source: Genes (Basel). 2022 Nov 29;13(12):2243. doi: 10.3390/genes13122243 (PMC9778007; doi:10.3390/genes13122243)
Supplement: Supplementary file 1 [file genes-13-02243-s001.zip › Supplemental Table S3.pdf]

Supplemental Table S3. Chromosome and location of 17 DE-NRGs.

Genes	GRCh38.p14		T2T-CHM13v2.0	
	Chr	Location	Chr	Location
H2AC18	1	NC_000001.11	1	NC_060925.1
TNFSF10	3	NC_000003.12	3	NC_060927.1
HSP90AB1	6	NC_000006.12	6	NC_060930.1
HMGB1	13	NC_000013.11	13	NC_060937.1
CASP1	11	NC_000011.10	11	NC_060935.1
H2AC8	6	NC_000006.12	6	NC_060930.1
SLC25A4	4	NC_000004.12	4	NC_060928.1
TRAF2	9	NC_000009.12	9	NC_060933.1
H2AC20	1	NC_000001.11	1	NC_060925.1
BID	22	NC_000022.11	22	NC_060946.1
H2AC21	1	NC_000001.11	1	NC_060925.1
MAPK10	4	NC_000004.12	4	NC_060928.1
ZBP1	20	NC_000020.11	20	NC_060944.1
TNFAIP3	6	NC_000006.12	6	NC_060930.1
PPIA	7	NC_000007.14	7	NC_060931.1
FADD	11	NC_000011.10	11	NC_060935.1
JAK3	19	NC_000019.10	19	NC_060943.1
